# Utilization of partograph and associated factors among obstetric caregivers in Ethiopia: a systematic review and meta-analysis

**DOI:** 10.3389/fgwh.2025.1339685

**Published:** 2025-01-27

**Authors:** Mulat Ayele, Eyob Shitie Lake, Gizachew Yilak, Getinet Kumie, Biruk Beletew Abate, Alemu Birara Zemariam, Befkad Derese Tilahun

**Affiliations:** ^1^ Department of Midwifery, College of Health Science, Woldia University, Woldia, Ethiopia; ^2^ Department of Nursing, College of Health Science, Woldia University, Woldia, Ethiopia; ^3^ Department of Medical Laboratory Sciences, College of Health Science, Woldia University, Woldia, Ethiopia; ^4^ College of Medicine and Health Sciences, Woldia University, Woldia, Ethiopia; ^5^ Department of Pediatrics and Child Health Nursing, School of Nursing, College of Health Science, Woldia University, Woldia, Ethiopia

**Keywords:** partograph, partograph utilization, systematic review, systematic review and meta-analysis, Ethiopia

## Abstract

**Introduction:**

Effective use of the partograph is crucial in identifying abnormal labor (prolonged and obstructed labor) and taking appropriate actions. However, in Ethiopia, the prevalence of obstructed labor is much higher at 11.8% and contributes to 14.4% of the total maternal deaths due to underutilization of the partograph by obstetric caregivers. Although a previous systematic review and meta-analysis (SRMA) had been conducted, they were not representative on a national level as they included a limited number of studies and did not evaluate the current prevalence of partograph utilization beyond July 2019. Since then, there have been inconsistent studies assessing the proportion of partograph utilization and its associated factors. Therefore, this systematic review and meta-analysis aimed to estimate the pooled prevalence of partograph utilization and its associated factors in Ethiopia.

**Method:**

Comprehensive literature searches were conducted in PubMed, Google Scholar, and HINARI from 1 September 2013 to 23 October 2023. A random-effects model was used to estimate pooled prevalence and adjusted odds ratio. Stata (version 11.0) was used to analyze the data. Cochrane *I*^2^ statistics were computed to assess heterogeneity among studies. A subgroup analysis was done based on the study region to minimize underlying heterogeneity. Funnel plot and Eggers test were conducted to assess publication bias.

**Result:**

Overall, 661 articles were retrieved, and finally, 23 studies were included in this systematic review, including 7,649 participants. The pooled prevalence of partograph utilization was 54.92% (95% CI: 43.38–66.45). The subgroup analysis showed that partograph utilization was highest in the Dire Dawa region and lowest in the Amhara region. Factors such as partograph training [adjusted odds ratio (AOR) = 3.63, 95% CI: 2.57–5.25], good knowledge about partograph (AOR = 2.63, 95% CI: 1.62–4.26), a favorable attitude toward partograph (AOR = 1.95, 95% CI: 1.35–2.82), partograph availability (AOR = 0.89, 95% CI: 2.24–6.61), and being in the midwifery profession (AOR = 0.09, 95% CI: 1.78–5.25) were significantly associated with partograph utilization.

**Conclusion:**

The pooled prevalence of partograph utilization in Ethiopia was low. Partograph training, good knowledge about partograph, favorable attitude toward partograph, partograph availability in the health facility, and being in the midwifery profession were significantly associated with partograph utilization.

**Systematic Review Registration:**

https://www.crd.york.ac.uk/prospero/, identifier (CRD42023475818).

## Introduction

The partograph is a standardized tool used to monitor the health status of both the mother and fetus during labor. It consists of three components: the fetal component, which monitors the fetal condition; the labor progress component, which tracks the progress of labor; and the maternal component, which assesses the mother's condition. The World Health Organization (WHO) recommends the use of the partograph to detect any abnormalities and aid in timely decision-making regarding the continuation of labor, augmentation, or the need for a cesarean delivery, starting from the active first stage of labor or when the cervix is dilated to 4 cm (1). By providing a visual overview of it, the partograph enables healthcare professionals to identify and diagnose abnormal labor progression, including prolonged and obstructed labor, which are major causes of maternal and neonatal deaths in developing countries, including Ethiopia (2). Globally, it is estimated that obstructed labor occurs in 5% of pregnancies and accounts for approximately 8% of maternal deaths (3, 4). However, in Ethiopia, the prevalence of obstructed labor is much higher at 11.8% and contributes to 14.4% of total maternal deaths (5, 6). Prolonged labor is also a leading cause of maternal and newborn deaths in developing countries. Therefore, the effective use of the partograph is crucial in identifying abnormal labor and taking appropriate actions (2). Research has shown that the utilization of the partograph can lead to significant improvements in labor outcomes. For example, its use has been associated with a reduction in the rate of prolonged labor, the proportion of labor requiring augmentation, emergency cesarean deliveries, and stillbirths (1, 7). The partograph includes a specific line that indicates the presence of difficulties such as slow labor progress, prolonged labor, fetal distress, obstructed labor, and ruptured uterus or when these issues should be addressed. Early detection of protracted or obstructed labor greatly aids in preventing complications such as postpartum hemorrhage, ruptured uterus, puerperal infections, and obstetric fistulas (6). However, there are barriers to the effective use of the partograph, particularly in low-resource settings. Challenges include limited resources, a shortage of healthcare personnel, low competency in using the tool, inadequate supervision, low acceptability of the tool, and a lack of functioning referral systems (8). Partograph utilization varies in different healthcare settings in Ethiopia, ranging from 6.9% in Jimma University Hospital (9) to 92.6% in eastern Ethiopia (10). Factors such as supervision, training on the partograph, knowledge about its use, the number of obstetric caregivers per shift, the working institution, and attitudes toward its utilization have been identified as factors influencing its utilization (11–15). Although a previous systematic review and meta-analysis (SRMA) have been conducted, they were not representative on a national level as they included a limited number of studies and did not evaluate the current prevalence of partograph utilization beyond July 2019 (16). Since then, there have been inconsistent studies assessing the proportion of partograph utilization and its associated factors. Therefore, this systematic review and meta-analysis aimed to estimate the pooled prevalence of partograph utilization and its associated factors in Ethiopia.

## Methods

### Study design and setting

A systematic review and meta-analysis (SRMA) were conducted to assess the partograph utilization in Ethiopians among obstetric caregivers who were in the process of labor follow-up. The study adhered to the Preferred Reporting Items for Systematic Review and Meta-Analysis (PRISMA) guidelines, which consist of checklists that provide guidance for conducting and reporting systematic reviews and meta-analyses (Supplementary Table S1). These guidelines aim to enhance transparency and accuracy in reviews conducted across various disciplines, including medicine (17). Ethiopia, classified as a low-income country, is located in the Horn of Africa and is projected to have a population of 123.4 million in 2022, 133.5 million in 2032, and 1,71.8 million in 2,050. From an administrative perspective, Ethiopia is divided into 11 regions and two city administrations. The regions are further subdivided into zones, and zones are further divided into districts. Finally, districts are divided into kebeles, which represent the smallest administrative divisions and typically have a population ranging from 2,000 to 3,500 residents.

### Search strategies and sources of information

We conducted a search in the PROSPERO database (https://www.crd.york.ac.uk/prospero/) to determine if any recently published or ongoing projects exist on the same topic, to avoid unnecessary duplication. Our search revealed that there were no ongoing or published articles related to this specific topic. Therefore, we registered this systematic review and meta-analysis in the PROSPERO database with the ID number CRD42023475818. To gather relevant articles, we conducted a comprehensive literature search using international databases such as PubMed, Google Scholar, Scopus, Web of Science, and HINARI. The search terms we used in this SRMA were as follows: “Prevalence” OR “epidemiology” OR “proportion” AND “associated factor” OR “factors” AND “utilization” OR “utilization of partograph” OR “partograph” AND “obstetric care givers” OR “obstetric care providers” AND “Ethiopia.”

### Inclusion criteria

This systematic review and meta-analysis focused on articles that met the specific criteria. We included studies that reported the prevalence or proportion of partograph utilization and the associated factors. Both gray literature and published articles written in the English language were considered. Specifically, we looked for cross-sectional studies that reported the prevalence or proportion of partograph utilization and its associated factors. The time frame for inclusion ranged from 1 September 2013 to 23 October 2023.

Since the SRMA was published on the topic in 2019, there have been inconsistent primary studies conducted in different parts of Ethiopia. This indicates that the SRMA conducted in 2019 does not show the current national figure of parthograph utilization. Therefore, we conducted an SRMA to estimate the current and most recent utilization level of partograph on studies published from 1 September 2013 to 23 October 2023.

### Exclusion criteria

In our selection process, we excluded articles that did not have full abstracts or complete texts available. We also excluded case reports, case studies, systematic reviews, meta-analyses, and articles that did not report on the outcome of interest. These exclusion criteria were applied to ensure that we included only relevant and complete studies in our systematic review and meta-analysis.

### Outcome of measurement

The first outcome of this systematic review and meta-analysis study focused on partograph utilization. The second outcome of this study aimed to identify the associated factors of partograph utilization.

### Data extraction

The datasets were exported to Mendeley Reference Manager, and from there, they were transferred to a Microsoft Excel spreadsheet for further analysis. The first step in the analysis process involved removing any duplicate data from the review. To ensure accurate data extraction, three authors (MA, EL, and BA) independently extracted all the relevant data using a standardized Joanna Briggs Institute (JBI) data extraction format. In cases where there were disagreements between the reviewers, a second team of reviewers (GY, BT, and AZ) was involved to resolve the discrepancies. The resolution process involved critical discussions and evaluations of the articles by an independent group of reviewers. The following information was extracted from the articles: author names, sample size, publication year, study area, region, study design, prevalence of partograph utilization, and adjusted odds ratios with a 95% confidence interval for factors associated with partograph utilization. By following this systematic data extraction process, the study aimed to ensure consistency and accuracy in capturing the relevant information from the selected articles.

### Quality assessment

To assess the quality of the included studies, the Newcastle-Ottawa Quality Assessment Scale (NOS) was used for both cross-sectional and case–control study designs (18) (Supplementary Table S2). Three authors (MA, BT, and GY) were responsible for independently assessing the quality of each study. The assessment covered various aspects, including methodological quality, sample selection, sample size, comparability, outcome assessment, and statistical analysis of the study.

In cases where there were disagreements among the three authors during the quality assessment, three additional authors (EL, GG, and BA) were involved. The disagreements were discussed and resolved through thorough deliberation and consensus among the authors. This process ensured that the quality assessment was conducted in a rigorous and comprehensive manner, considering multiple perspectives and expertise among the author team.

### Data processing and analysis

The extracted data in Microsoft Excel spreadsheet format was imported into STATA version 11 for analysis. A random-effects model was employed to estimate the pooled prevalence of partograph utilization among obstetric caregivers in Ethiopia. To assess the heterogeneity among the included studies, Cochrane *I*^2^ statistics were calculated. The *I*^2^ value provides an indication of the percentage of variation across studies that can be attributed to heterogeneity rather than chance.

Based on the *I*^2^ values, heterogeneity was categorized as follows: 0%–40% indicating mild heterogeneity, 40%–70% indicating moderate heterogeneity, and 70%–100% indicating considerable heterogeneity (19). A subgroup analysis was conducted based on the study region to explore potential variations in the prevalence of partograph utilization. To examine the potential risk of publication bias, funnel plots and Egger's test were performed (20). The *p*-value of the Egger's test (0.345) indicated the absence of publication bias, as it was greater than the significance level of 0.05.

The pooled prevalence and pooled adjusted odds ratios (OR) for factors associated with partograph utilization among obstetric caregivers were presented in a forest plot format. The forest plot included the point estimates of prevalence and OR, along with their corresponding 95% confidence intervals (CI). This format allowed for a visual representation of the pooled results and provided a comprehensive overview of the estimates and their precision.

### Subgroup and sensitivity analyses

Subgroup analyses were performed based on the study region to examine potential variations in the prevalence of partograph utilization among obstetric caregivers in different regions of Ethiopia. This analysis aimed to explore whether the prevalence estimates differed significantly across different geographical areas.

In addition, sensitivity analysis was conducted to assess the stability and robustness of the pooled estimates to outliers and the potential impact of individual studies on the overall results. Sensitivity analysis helps evaluate the influence of individual studies on the overall findings by systematically excluding one study at a time and reanalyzing the data. This analysis allows for a better understanding of the potential impact of specific studies on the pooled estimates and the overall conclusions of the systematic review and meta-analysis.

## Result

### Characteristics of the included studies

**Figure 1:**
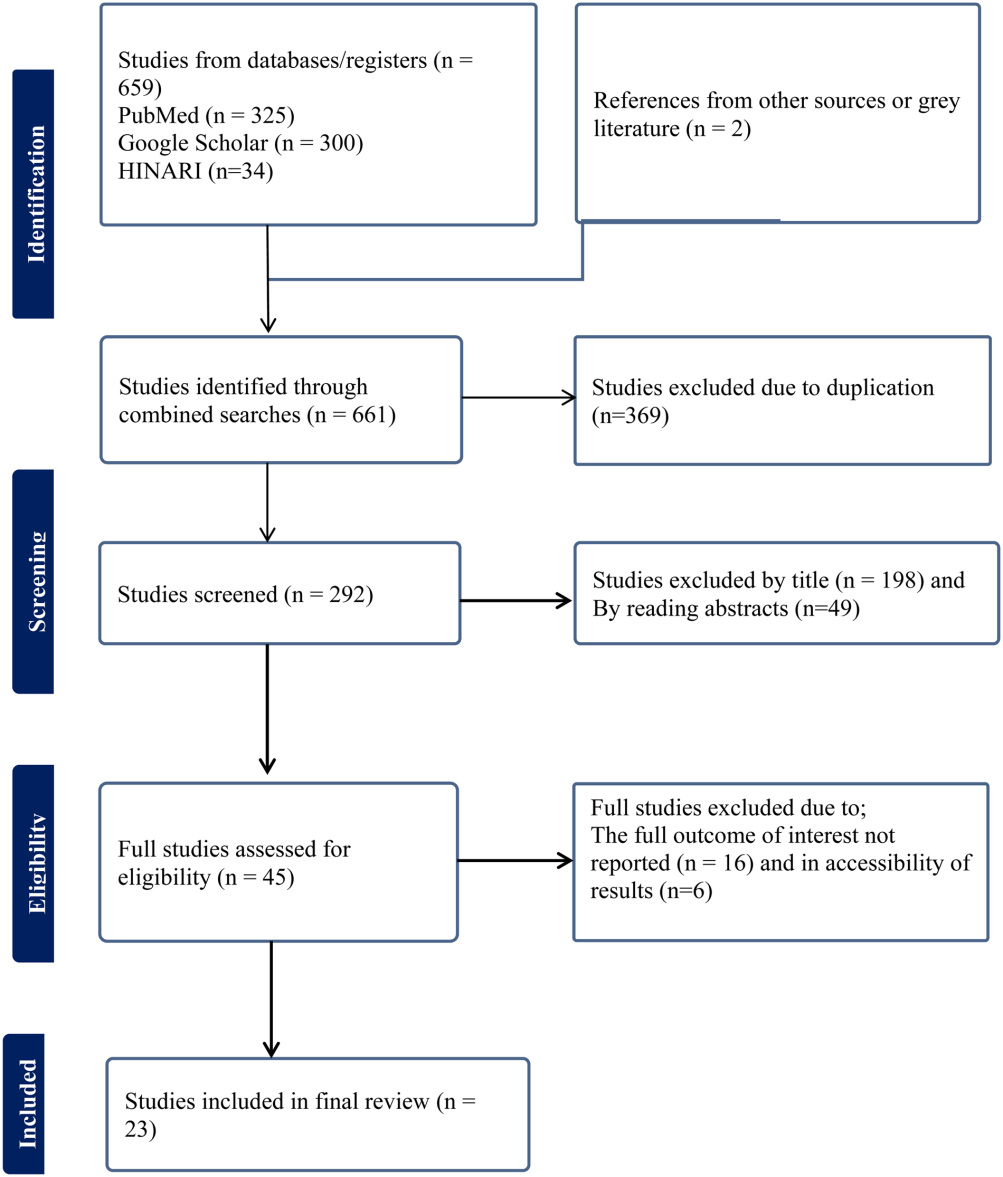
Flowchart of selection for systematic review and meta-analysis on partograph utilization among obstetric caregivers in Ethiopia, 2023.

A total of 661 articles were found using our search strategy in HINARI, Google Scholar, and PubMed databases and repositories since 2013. After removing duplicates (369), 292 articles remained. Subsequently, 198 articles were excluded based on reviewing the titles, and another 49 were excluded based on the abstracts. Full-text papers were then accessed and evaluated for inclusion criteria, leading to the exclusion of 22 more articles based on the aforementioned criteria. Therefore, 23 papers were eligible for inclusion in the final systematic review and meta-analysis (Figure 1). Of the included studies in this SRMA, seven were conducted in Oromia (2, 9, 14, 21–24), six in South Nation Nationality and People Region (SNNPR) (11, 25–29), three in Amhara (30–32), two in Addis Ababa (12, 33) and Tigray (34, 35), and one in Somali (36), Sidama (37), and Dire Dawa (10). All 23 included studies were cross-sectional, involving a total of 7,649 participants, ranging from 127 to 594 participants per study. The studies reported partograph utilization rates of 6.9% to 92.6%. In terms of the quality of included studies, the Newcastle-Ottawa Quality Assessment Scale score for all included studies ranged from 8 to 9, indicating good quality (Table 1).

**Table 1:** Characteristics of included studies in the systematic review and meta-analysis on utilization of partograph among obstetric caregivers in Ethiopia, 2023.

S. No	Author, year	Region	Study design	Sample size	Prevalence of partograph utilization (%)	Quality
1	Hagos, Teka, and Degu, 2020	Addis Ababa	Cross-sectional	594	69	Good
2	Yisma et al., 2013	Addis Ababa	Cross-sectional	403	57.3	Good
3	Markos and Bogale, 2015	Oromia	Cross-sectional	401	70.2	Good
4	Hailu et al., 2018	Tigray	Cross-sectional	198	73.3	Good
5	Abate, Mph, and Temesgen, 2022	Amhara	Cross-sectional	267	28.84	Good
6	Mezmur, Semahegn, and Tegegne, 2017	Dire Dawa	Cross-sectional	441	92.6	Good
7	Haile et al., 2020	SNNPR	Cross-sectional	436	54.4	Good
8	Negash and Alelgn, 2022	Sidama	Cross-sectional	405	58.4	Good
9	Tesfaye and Chanie, 2023	Oromia	Cross-sectional	250	32.8	Good
10	Wakgari et al., 2015	Amhara	Cross-sectional	403	40.2	Good
11	Eshetu, Hussen, and Dulla, 2017	SNNPR	Cross-sectional	286	50.7	Good
12	Mekonen et al., 2022	Somali	Cross-sectional	235	41	Good
13	Tilahun et al., 2021	SNNPR	Cross-sectional	393	43	Good
14	Ayele, Tadesse, and Haile, 2023	SNNPR	Cross-sectional	410	55.1	Good
15	Gebreslassie et al., 2019	Tigray	Cross-sectional	406	83	Good
16	Bedada, Huluka, and Bulto, 2020)	Oromia	Cross-sectional	322	31.1	Good
17	Getu et al., 2020	SNNPR	Cross-sectional	442	73.6	Good
18	Markos, Arba, and Paulos, 2020	SNNPR	Cross-sectional	269	71.7	Good
19	Kitila, 2014	Oromia	Cross-sectional	340	6.9	Good
20	Regasa, Tilahun, and Adem, 2018	Oromia	Cross-sectional	202	89.1	Good
21	Bekele et al., 2017	Oromia	Cross-sectional	127	26	Good
22	Abebe et al., 2013	Amhara	Cross-sectional	160	29.3	Good
23	Willi and Sciences, 2017	Oromia	Cross-sectional	259	84.6	Good

### Pooled prevalence of partograph utilization among obstetric caregivers in Ethiopia

**Figure 2:**
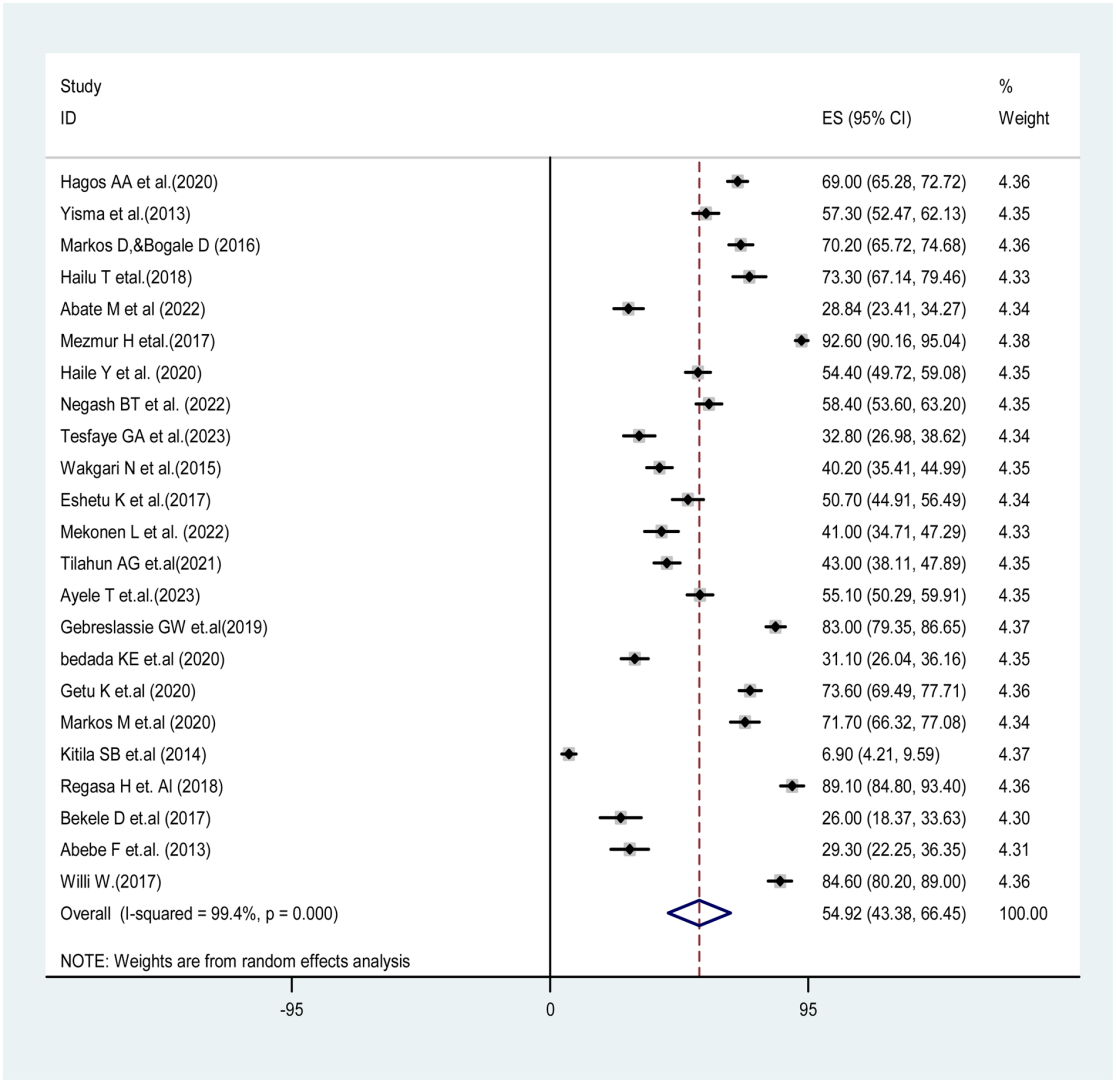
The pooled prevalence of partograph utilization among obstetric caregivers in Ethiopia, 2023.

The pooled prevalence of partograph utilization among obstetric caregivers in Ethiopia was 54.92% (CI: 43.38–66.45), with the Cochrane heterogeneity index (*I*^2^ = 99.4%, *p* = 0.000) indicating substantial heterogeneity among different studies (*I*^2^ > 70%). Therefore, we used the random-effects model to address the issue of heterogeneity among the included studies. Additionally, we considered subgroup analysis as a potential way of addressing heterogeneity (Figure 2).

### Subgroup analysis of partograph utilization among obstetric care providers in Ethiopia

**Figure 3:**
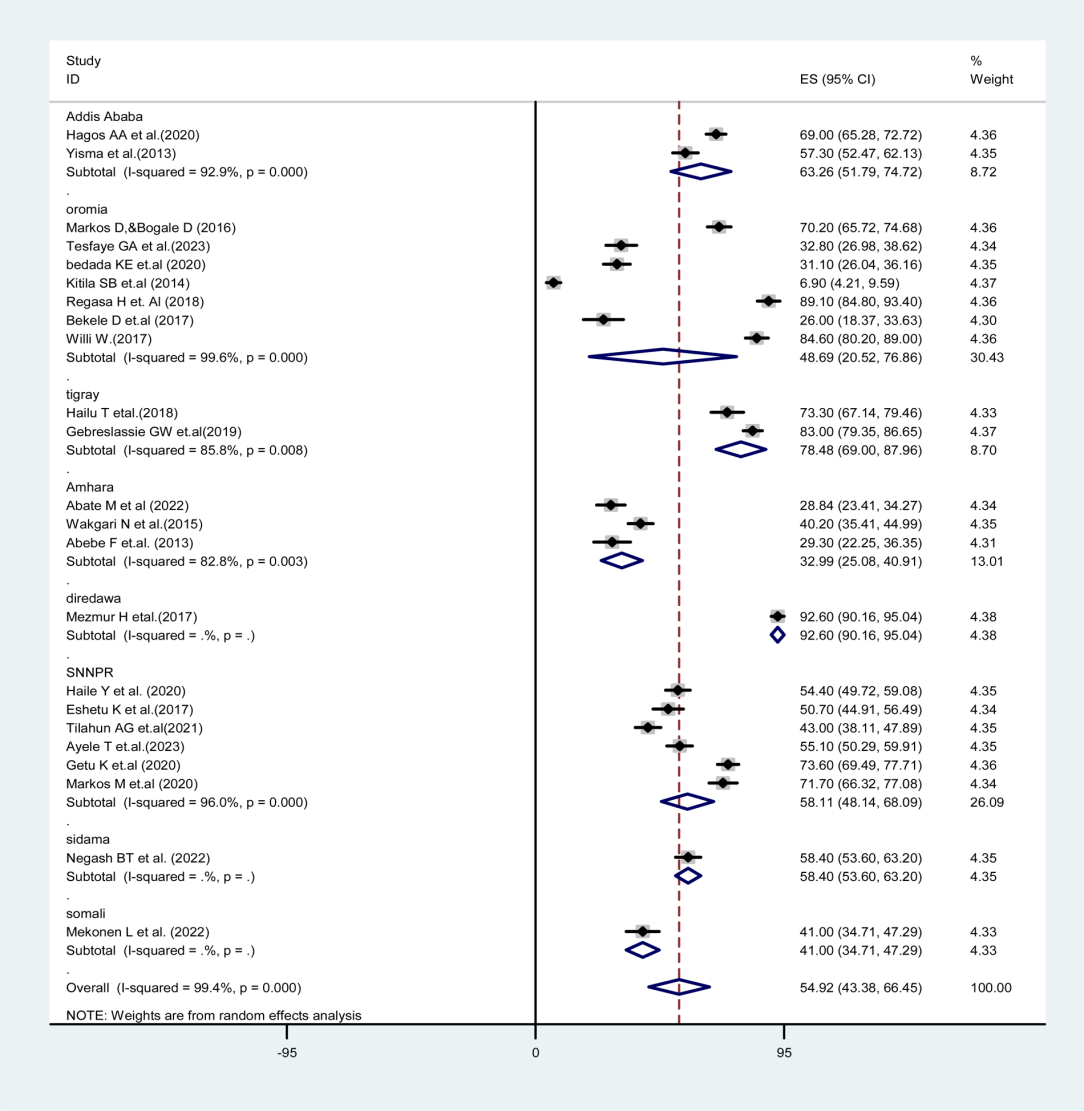
Forest plot showing the subgroup analysis of partograph utilization among obstetric caregivers in Ethiopia, 2023.

In this systematic review and meta-analysis, the finding of subgroup analysis by region showed that the pooled prevalence of partograph utilization among obstetric caregivers was lowest in the Amhara region [32.99%; 95% CI: (25.08–40.91), *I*^2^ = 82.8%, *p* = 0.003], while it was highest in the Dire Dawa region (92.6%; 95% CI: (90.16–95.04), *I*^2^ = .%, *p* = .). These findings suggest regional variations in the prevalence of partograph utilization in Ethiopia, with lower rates observed in the Amhara region and higher rates in the Dire Dawa region. These differences may reflect variations in healthcare practices, resource availability, or other regional factors influencing partograph utilization (Figure 3).

### Publication bias

**Figure 4:**
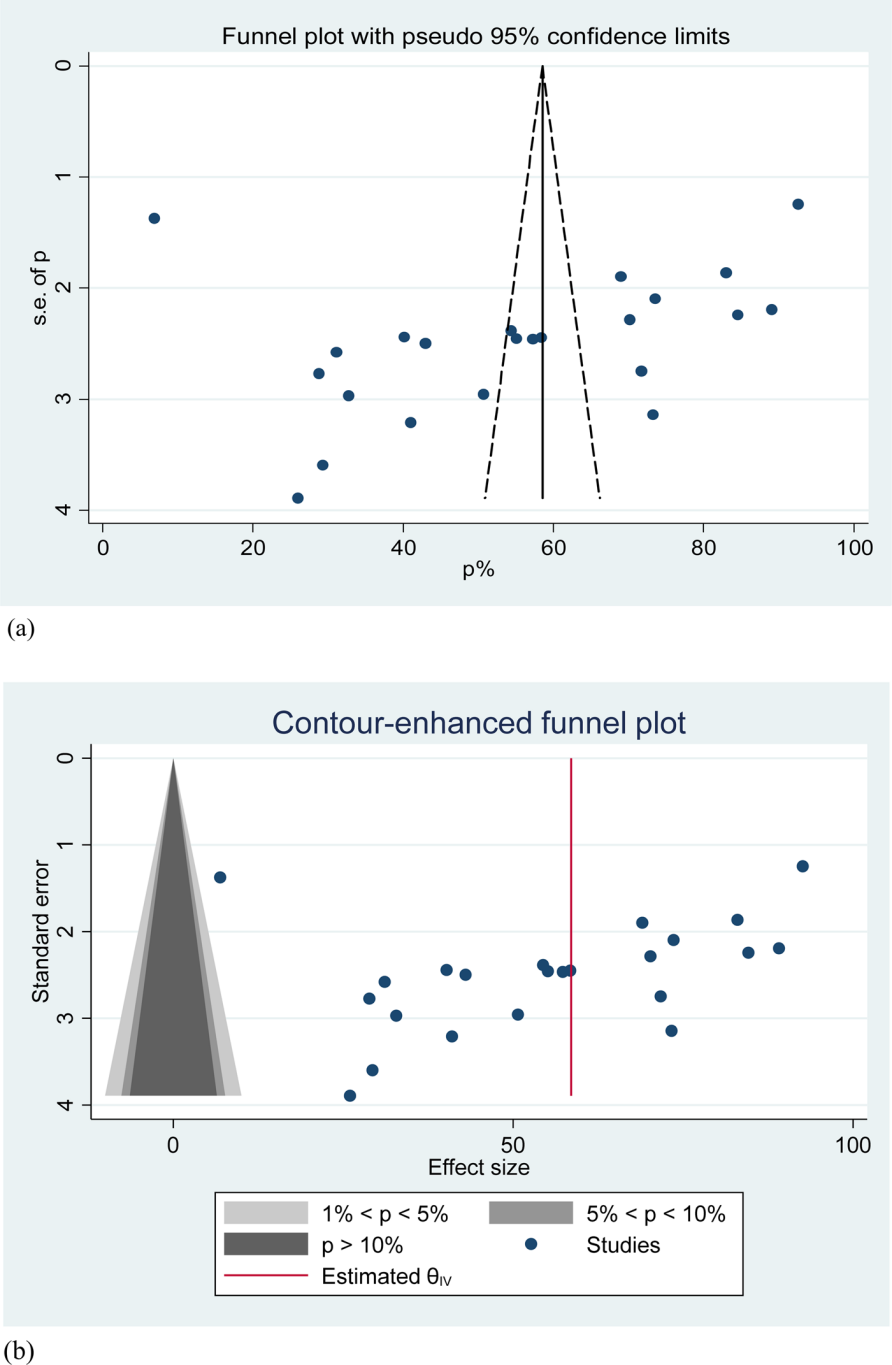
**(a)** Funnel plot for assessing publication bias of the prevalence of partograph utilization among obstetric caregivers in Ethiopia, 2023. **(b)** Contour funnel plot for assessing the publication bias of the prevalence of partograph utilization among obstetric caregivers in Ethiopia, 2023.

In this systematic review and meta-analysis, the presence of publication bias was assessed using a funnel plot and contour-enhancing funnel plot, which visually inspected the asymmetry of the distribution of partograph utilization studies. Additionally, Egger's regression test was conducted, resulting in a *p*-value of 0.345 (*p* > 0.05), indicating the absence of publication bias (Figures 4a,b).

### Sensitivity analysis

**Figure 5:**
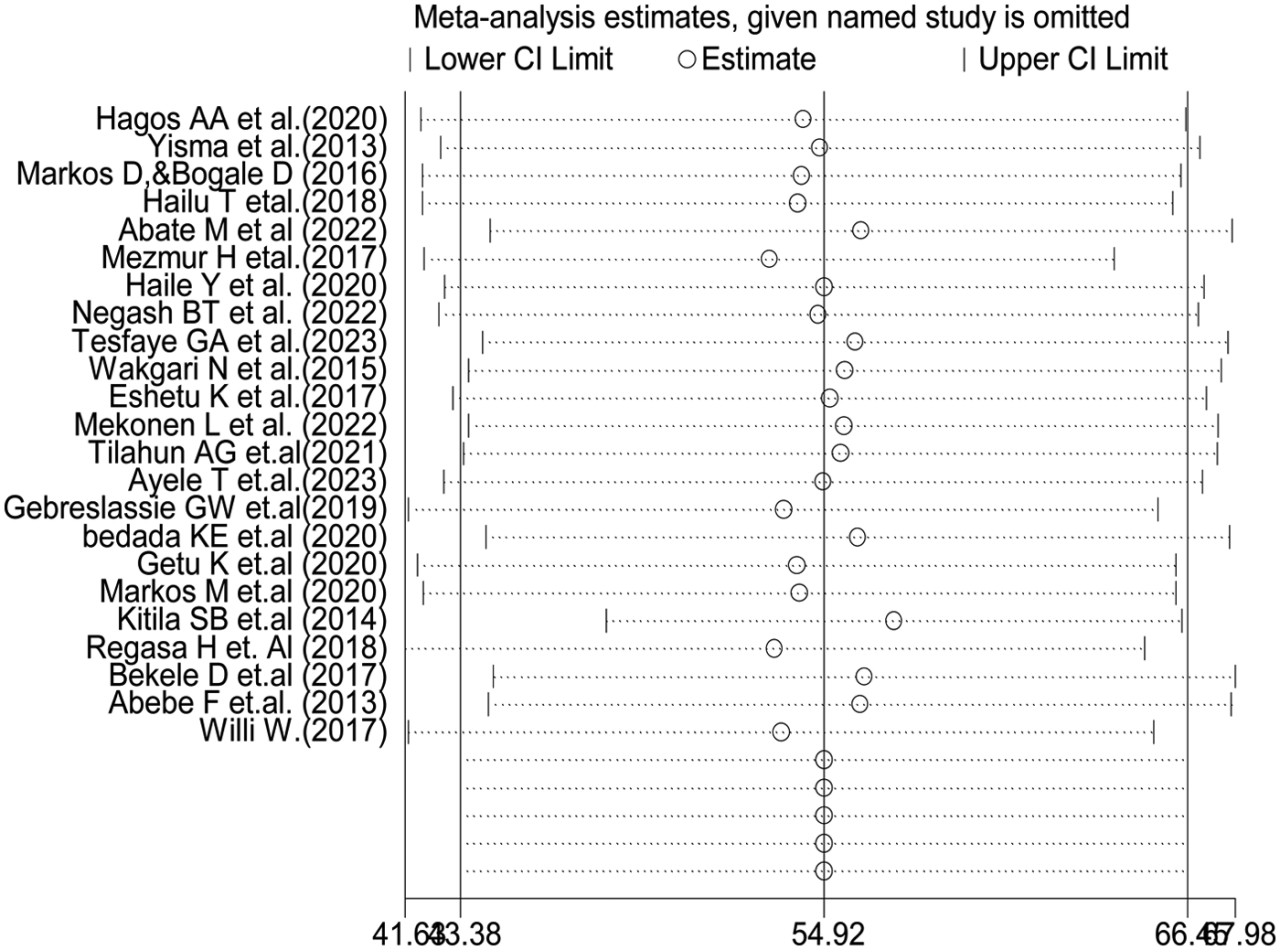
Sensitivity analysis of partograph utilization among obstetric caregivers in Ethiopia, 2023.

The results of the random-effects model indicated that the overall pooled prevalence of partograph utilization among obstetric care providers in Ethiopia was influenced by a specific individual study (Figure 5).

### Determinants of partograph utilization among obstetric caregivers in Ethiopia

In our systematic review and meta-analysis, several factors were found to be significantly associated with partograph utilization among obstetric care providers in Ethiopia. These factors include receiving partograph training, having good knowledge about the partograph, maintaining a favorable attitude toward partograph utilization, partograph availability, and being in the midwifery profession.

Obstetric care providers who were trained in using the partograph were 3.63 times more likely to utilize the partograph during labor follow-up, starting from the active first stage of labor, compared to their counterparts [adjusted odds ratio (AOR) = 3.63, 95% CI: 2.57–5.25]. Obstetric care providers with good knowledge were 2.63 times more likely to use partograph than those with less knowledge (AOR = 2.63, 95% CI: 1.62–4.26). Similarly, obstetric care providers with a favorable attitude were almost twice as likely to use the partograph compared to those with an unfavorable attitude toward partograph utilization (AOR = 1.95, 95% CI: 1.35–2.82).

The study revealed that participants who had access to the partograph in their health facility were almost 3.89 times more likely to utilize the partograph compared to those without access (AOR = 3.89, 95% CI: 2.24–6.61). Additionally, being in the midwifery profession was associated with almost three times higher odds of using partograph compared to other professions (AOR = 3.09, 95% CI: 1.78–5.25) (Table 2).

**Table 2:** Factors associated with partograph utilization among obstetric caregivers in Ethiopia, 2023.

Variable	Authors	AOR	95%CI	Pooled AOR	95% CI of pooled AOR
Receiving partograph training	Hagos, Teka, and Degu, 2020	2.4	1.5–3.6	3.63	2.57–5.25
	Yisma et al., 2013	2.56	1.03–6.25		
	Markos and Bogale, 2015	2.94	1.15–7.54		
	Hailu et al., 2018	5.6	1.1–28.5		
	Abate, Mph, and Temesgen, 2022	3.63	1.45–9.09		
	Mezmur, Semahegn, and Tegegne, 2017	3.1	1.35–4.98		
	Haile et al., 2020	7.06	4.3–11.37		
	Negash and Alelgn, 2022	1.9	1.1–3.3		
	Tesfaye and Chanie, 2023	2.21	1.19–4.11		
	Wakgari et al., 2015	2.86	1.69–4.86		
	Mekonen et al., 2022	15.46	6.95–34.42		
	Tilahun et al., 2021	7.83	4.54–13.5		
	Ayele, Tadesse, and Haile, 2023	1	0.4–2.4		
	Gebreslassie et al., 2019	1.173	0.6–2.33		
	Bedada, Huluka, and Bulto, 2020	.94	1.99–7.78		
	Markos, Arba, and Paulos, 2020	6.25	2.33–16.66		
	Willi and Sciences, 2017	4	0.4–21.7		
Good knowledge about partograph	Hagos, Teka, and Degu, 2020	1.6	1.1–2.5	2.63	1.62–4.26
	Abate, Mph, and Temesgen, 2022	1.68	1.21–3.02		
	Wakgari et al., 2015	2.12	1.3–3.9		
	Haile et al., 2020	0.47	0.284–0.76		
	Negash and Alelgn, 2022	3.79	2.05–7.03		
	Mekonen et al., 2022	6.9	2.62–18.18		
	Tilahun et al., 2021	5.84	3.27–10.44		
	Ayele, Tadesse, and Haile, 2023	2	1.2–3.5		
	Getu et al., 2020	1.82	0.79–4.16		
	Markos and Bogale, 2015	3.35	1.61–6.97		
	Bekele et al., 2017	5	1.49–1.56		
	Willi and Sciences, 2017	7	2.8–21.8		
Favorable attitude	Abate, Mph, and Temesgen, 2022	2	1.25–5.32	1.95	1.35–2.82
	Haile et al., 2020	1.8	1.12–2.97		
	Negash and Alelgn, 2022	0.9	0.32–2.55		
	Wakgari et al., 2015	2.35	1.14–4.87		
	Mekonen et al., 2022	2.99	1.25–7.14		
	Ayele, Tadesse, and Haile, 2023	3.7	1.7–7.7		
	Bedada, Huluka, and Bulto, 2020	2.48	1.23–5.02		
	Getu et al., 2020	0.54	0.24–1.18		
	Markos and Bogale, 2015	2.99	1.28–7		
Paragraph availability	Abate, Mph, and Temesgen, 2022	1.44	0.74–2.79	3.89	2.24–6.61
	Tesfaye and Chanie, 2023	4.19	2.12–8.29		
	Mekonen et al., 2022	4.63	1.7–12.64		
	Bedada, Huluka, and Bulto, 2020	5.23	1.69–16.22		
	Bekele et al., 2017	4.36	1.41–13.44		
	Willi and Sciences, 2017	8.8	2.8–27.6		
Midwife profession	Markos and Bogale, 2015	1.13	0.37–3.46	3.09	1.78–5.25
	Wakgari et al., 2015	8.13	2.67–24.78		
	Tilahun et al., 2021	2.7	1.52–4.76		
	Ayele, Tadesse, and Haile, 2023	3.4	1.2–9.4		
	Getu et al., 2020	4.7	1.8–12		
	Markos, Arba, and Paulos, 2020	0.38	0.06–2.32		
	Bekele et al., 2017	2.6	1.01–6.68		
	Willi and Sciences, 2017	13	2.6–66.2		

AOR is the odds ratio of the respective variable in each primary study. Pooled AOR is the point value of the odds ratio when we pool the AOR of primary studies by our analysis. 95% CI of pooled AOR is the 95% CI of the point value of pooled AOR that is the output of our analysis.

## Discussion

A continuous pictorial overview of labor through the partograph alerts midwives and obstetricians to identify and intervene in cases of abnormalities in maternal or fetal conditions, aiming to prevent prolonged labor, obstructed labor, and its complications (33, 38). In this systematic review and meta-analysis, the pooled prevalence of partograph utilization among obstetric caregivers was 54.92% (CI: 43.38–66.45). This finding aligns with a study done at a Regional Hospital in the eThekwini District, which a reported prevalence of 62% (38). However, our review finding was lower than the WHO recommendation, which recommends that all laboring women should be followed using partograph for the well-being of both the mother and the fetus (39). Similarly, this prevalence was lower than in studies conducted in Uganda (69.9%) (40), the Gambia (78%) (41), South Africa (79.4%) (42), and Gazi (89.9%) (43). This disparity can be attributed to differences in sample size, location, availability of the partograph, staffing level and experience, and variations in the implementation of WHO recommendations regarding partograph utilization.

On the other hand, our finding was higher than those of a study conducted in the 2016 National Emergency Obstetric and Newborn Care Survey of Ethiopia (21.5%) (44), Nigeria, (32.3%) (45), and Tanzania (38.7%) (46) another study in Nigeria (22.2%) (47), Malawi (3.9%), Cameron (35%) (48), and northwest and southwest Cameron 32.4% (49). The disparity between these studies and our finding could be attributed to differences in sample size and study period. Our findings represent a pooled prevalence that includes all the latest studies from various regions of the country, while those studies were single-centered with fewer samples, leading to variations in the prevalence of partograph utilization. The variation between the 2016 National Emergency Obstetric and Newborn Care Survey of Ethiopia and our finding is due to difference in study period. Our review includes all studies conducted from 2013 to 2023, capturing changes in obstetric care providers' awareness, attitude, practice, and training regarding partograph utilization. In contrast, the 2016 national survey only includes data from the 12 months preceding the survey (44). This makes difference in prevalence of partograph utilization among obstetric caregivers.

This systematic review and meta-analysis also identified factors that showed a significant association with partograph utilization among obstetric caregivers. Specifically, obstetric care providers who underwent partograph training were 3.63 times more likely to use partograph during labor follow-up starting from the active first stage of labor than their counterparts. This is supported by previous studies done in Gazi (43), Malawi (50), and Uganda (40). This is because training on partograph utilization and its importance enhances knowledge, improves attitude, and enhances skills, thereby increasing partograph utilization.

The current review showed that obstetric care providers with good knowledge were 2.63 times more likely to use the partograph than those with less knowledge. This finding is consistent with previous studies done in Nigeria (51, 52), Enugu Metropolis (53), and Cameron (49). This correlation may be attributed to the fact that effectively using the partograph requires knowledge about when to initiate it, its components, the procedures for its use, and its significance in reducing instances of prolonged and obstructed labor. Therefore, individuals with a strong understanding of these aspects are more likely to utilize the partograph effectively as a tool for monitoring labor, thereby reducing the risk of maternal and neonatal deaths related to prolonged and obstructed labor (53).

In this SRMA, obstetric care providers who had favorable attitudes toward partograph utilization were almost two times more likely to use partograph compared to those who had unfavorable attitudes toward partograph utilization. This finding is consistent with the findings of a previous study conducted in Gazi (43). The possible reason might be that those who had a positive attitude toward the use of partograph were likely committed to using it to monitor feto-maternal conditions starting from the active first stage of labor.

Regarding partograph availability, the study revealed that those participants who had partograph in their health facility were almost four times more likely to utilize partograph compared to those who had no partograph. This finding is supported by a study conducted in Nigeria (51, 52) and Sokoto metropolis in Nigeria (47). This might be due to the availability of partograph which is vital in motivating and encouraging obstetric caregivers to utilize it as a labor monitoring tool from the active first stage of labor.

Being in the midwifery profession was associated with almost three times higher odds of using the partograph compared to other professions. This finding is supported by a previous study conducted in South Africa (54). This could be due to midwives being primarily assigned to labor and intrapartum wards compared to other professionals, which increases their likelihood of receiving training in partograph utilization and motivates them to use the partograph as a labor monitoring tool for making informed decisions.

### Limitations and strengths of the study

One of the strengths of this review is that it incorporates both published and unpublished studies, which helps reduce the risk of publication bias. Additionally, a significant number of studies from various regions were included, enhancing the representativeness of the findings. The limitation of this systematic review and meta-analysis is the lack of similar reviews conducted in other countries which makes it challenging to directly compare our findings with those of other studies, necessitating comparisons primarily with individual primary studies.

## Conclusion

The prevalence of partograph utilization among obstetric caregivers in Ethiopia was low. Several factors, including on-site partograph training, knowledge about partograph, attitude toward partograph utilization, paragraph availability in the health facility, and being in the midwifery profession were significantly associated with partograph utilization. Based on these findings, on-site training about partograph utilization and its importance related to feto-maternal outcomes should be given to obstetric care providers to increase the knowledge, attitude, and practice of partograph utilization simultaneously. This decreases prolonged and obstructed labor-related maternal and neonatal morbidity and mortality in Ethiopia.

## Data Availability

The original contributions presented in the study are included in the article/Supplementary Material, further inquiries can be directed to the corresponding author.
