# Supplementary material for: Utilization of partograph and associated factors among obstetric caregivers in Ethiopia: a systematic review and meta-analysis
Source: Front Glob Womens Health. 2025 Jan 27;6:1339685. doi: 10.3389/fgwh.2025.1339685 (PMC11808142; doi:10.3389/fgwh.2025.1339685)
Supplement: Supplementary file 2 [file Table2.docx]

| **Supplementary table 2: Newcastle-Ottawa Quality Assessment Scale**  **Tble1: Newcastle-Ottawa Quality Assessment Scale for cross sectional studies used in the systematic review and meta-analysis 2023** | | | | | | | | |
| --- | --- | --- | --- | --- | --- | --- | --- | --- |
|  | Selection | | | | Comparability | Outcome | | Total score |
| Authors | Representativeness s (1) | Sample size (1) | Non-respondents (1) | Ascertainment of the exposure (risk factor) (2) | The subjects in different outcome groups are comparable, based on the study design or analysis. confounding factors are controlled (1) | Assessment of the outcome (2) | Statistical test (1) |  |
| Hagos AA et al.(13) | 1 | 1 | 1 | 2 | 1 | 2 | 1 | 9 |
| Yisma et al.(43) | 1 | 1 | 1 | 2 | 1 | 2 | 1 | 9 |
| Markos D,&Bogale D (7) | 1 | 1 | 1 | 2 | 1 | 2 | 1 | 9 |
| Hailu T et al.(44) | 1 | 0 | 1 | 2 | 1 | 2 | 1 | 8 |
| Abate M et al. (45) | 1 | 1 | 1 | 2 | 1 | 2 | 1 | 9 |
| Mezmur H etal**.**(46) | 1 | 1 | 1 | 2 | 1 | 2 | 1 | 9 |
| Haile Y et al.(47) | 1 | 1 | 1 | 2 | 1 | 2 | 1 | 9 |
| Negash BT et al. (48) | 1 | 1 | 1 | 2 | 1 | 2 | 1 | 9 |
| Tesfaye GA et al.(49) | 1 | 1 | 1 | 2 | 1 | 2 | 1 | 9 |
| Wakgari N et al.(50) | 1 | 1 | 1 | 2 | 1 | 2 | 1 | 9 |
| Eshetu K et al. (12) | 1 | 1 | 1 | 2 | 1 | 2 | 1 | 9 |
| Mekonen L et al. (51) | 1 | 1 | 1 | 2 | 1 | 2 | 1 | 9 |
| Tilahun AG et.al.(21) | 1 | 1 | 1 | 2 | 1 | 2 | 1 | 9 |
| Ayele T et.al.(52) | 1 | 1 | 1 | 2 | 1 | 2 | 1 | 9 |
| Gebreslassie GW et.al(22) | 1 | 1 | 1 | 2 | 1 | 2 | 1 | 9 |
| Bedada KE et.al (15) | 1 | 1 | 1 | 2 | 1 | 2 | 1 | 9 |
| Getu K et.al (53) | 1 | 1 | 1 | 2 | 1 | 2 | 1 | 9 |
| Markos M et.al (54) | 1 | 1 | 1 | 2 | 1 | 2 | 1 | 9 |
| Kitila SB et.al (10) | 1 | 1 | 1 | 2 | 1 | 2 | 1 | 9 |
| Regasa H et. al.(55) | 1 | 1 | 1 | 2 | 1 | 2 | 1 | 9 |
| Bekele D et.al.(23) | 1 | 0 | 1 | 2 | 1 | 2 | 1 | 8 |
| Abebe et.al. (56) | 1 | 0 | 1 | 2 | 1 | 2 | 1 | 8 |
| Willi W.(57) | 1 | 1 | 1 | 2 | 1 | 2 | 1 | 9 |

- The scoring process was made according to Newcastle-Ottawa Quality Assessment Scale adapted for cross sectional studies

**Selection: (Maximum 5 scores)**

**1) Representativeness of the cases:**

a) Truly representative of the HCC patients (consecutive or random sampling of cases). 1 score

b) Somewhat representative of the average in the HCC patients (non-random sampling) . 1 score

c) Selected demographic group of users. 0 score

d) No description of the sampling strategy. 0 score

**2) Sample size:**

a) Justified and satisfactory (≥ 400 HCC included). 1 score

b) Not justified (<400 HCC patients included). 0 score

**3) Non-Response rate**

a) The response rate is satisfactory (≥95%). 1 Score

b) The response rate is unsatisfactory (<95%), or no description. 0 Score

**4) Ascertainment of the screening/surveillance tool:**

a) Validated screening/surveillance tool. 2 scores

b) Non-validated screening/surveillance tool, but the tool is available or described. 1 score

c) No description of the measurement tool. 0 score

**Comparability: (Maximum 1 scores)**

1) **The potential confounders were investigated by subgroup analysis or multivariable analysis.**

a) The study investigates potential confounders. 1 score

b) The study does not investigate potential confounders. 0 score

**Outcome: (Maximum 3** scores**)**

**1) Assessment of the outcome:**

a) Independent blind assessment. 2 scores

b) Record linkage. 2 scores

c) Self report. 1 score

d) No description. 0 score

**2) Statistical test:**

a) The statistical test used to analyze the data is clearly described and appropriate. 1 score

b) The statistical test is not appropriate, not described or incomplete. 0 score
